# Association of a Common Variant at 10q26 and Benign Prostatic Hyperplasia Aggressiveness in Han Chinese Descent

**DOI:** 10.1155/2013/820849

**Published:** 2013-08-01

**Authors:** Xin Gu, Tao Huang, Ding Xu, Liujian Duan, Yang Jiao, Jian Kang, S. Lilly Zheng, Jianfeng Xu, Jielin Sun, Jun Qi

**Affiliations:** ^1^Department of Urology, Xinhua Hospital, Medical School of Shanghai Jiaotong University, Shanghai 200092, China; ^2^Center for Cancer Genomics, Wake Forest University School of Medicine, Winston-Salem, NC 27157, USA; ^3^Center for Genomics and Personalized Medicine Research, Wake Forest University School of Medicine, Winston-Salem, NC 27157, USA; ^4^Fudan-VARI Center for Genetic Epidemiology, Fudan University, Shanghai 200422, China

## Abstract

Recent studies reported that rs2252004 at 10q26 was significantly associated with prostate cancer (PCa) risk in a Japanese population and was subsequently confirmed in a Chinese population. We aimed to assess the relationship between this locus and risk/aggressiveness of benign prostatic hyperplasia (BPH). The current study included 426 BPH cases and 1,008 controls from Xinhua Hospital in Shanghai, China. All BPH patients were treated with **α**-adrenergic blockers and 5**α**-reductase inhibitors for at least 9 months. Associations between rs2252004 and BPH risk/aggressiveness were tested using logistic regression. Associations between rs2252004 and clinical parameters including International Prostate Symptom Score (IPSS), total prostate volume (TPV), total PSA (tPSA), and free PSA (fPSA) were evaluated by linear regression. Allele “A” in rs2252004 was significantly associated with increased risk for aggressiveness of BPH in a Chinese population (OR = 1.42, 95% CI: 1.04–1.96, *P* = 0.03). Patients with the genotype “A/A” (homozygous minor allele) had an increase of IPSS and TPV after treatment (*P* = 0.045 and 0.024, resp.). No association was observed between rs2252004, BPH risk, and baseline clinicopathological traits (All *P* > 0.05). Our study is the first to show that rs2252004 at 10q26 was associated with BPH aggressiveness and efficacy of BPH treatment.

## 1. Introduction

Benign prostate hyperplasia (BPH) is an independent disease with clinical symptoms similar to those of carcinoma of the prostrate (PCa), and the prevalence of histologically identifiable BPH is >50% for 60-year-old men and ~90% by age 85 years [1]. While symptomatic BPH is not life threatening, it has a severe impact on the quality of life and requires immediate therapeutic interventions.

While studies have shown that BPH causes significant morbidity, the etiology and determinants of severity of this condition remain poorly understood. According to epidemiological studies, 83.3% of PCa are associated with BPH, and 3–20% of patients who have undergone transurethral prostatectomy (TURP) or open prostatectomy for BPH subsequently develop PCa [2]. Although BPH is not considered to be a premalignant lesion or a precursor of PCa, studies have observed anatomic, pathologic, and epidemiological associations and genetic links between PCa and BPH [3]. 

Over 40 SNPs have been reported to contribute to PCa risk in different ethnicities [4–6]. Fifteen of these SNPs were associated with PCa risk in a Chinese population [7, 8]. The relationship between these SNPs and BPH was recently studied [9, 10]. Three SNPs: rs103294 at LILRA3, rs12621278 at 2q31, and rs339331 at 6q22, were significantly associated with BPH risk. In addition, rs12621278 and rs12653946 at 5p15 were significantly associated with aggressive BPH [9, 10]. These results suggest that similar genetic mechanisms may predispose to benign and malignant prostate disease.

A recent GWAS PCa study identified rs2252004 at 10q26 to be significantly associated with PCa risk at a genome-wide significant level (*P* = 1.98*E* − 8) in the Japanese population [11]. It was further confirmed to contribute to PCa risk in a Chinese population [12]. The 10q26 region has been reported to be a loss-of-heterozygosity site in many types of cancers, including prostate cancer [13, 14]. Our study aimed to evaluate the relationship between rs2252004 and BPH risk/aggressiveness in a Chinese population. 

## 2. Materials and Methods

### 2.1. Study Subjects

All cases were of Han Chinese descent. BPH cases were enrolled from the Department of Urology at Xinhua Hospital (Shanghai Jiao Tong University School of Medicine, China) from July 2010 to July 2012. Male volunteers from multiple communities in Shanghai, China, were recruited as controls, from April 2010 to November 2010. All participants gave informed consent, and the study was approved by Xinhua's Ethics Committee prior to involvement in this study.

The information we obtained from subjects included the International Prostate Symptom Score (IPSS), quality of life question (IPSS-Q1), postvoid residual volume (PVR), prostate size, serum prostate-specific antigen (PSA) level, liver and renal function, blood glucose level, and routine urine examination.

BPH cases included in this study must meet the following criteria: benign prostatic enlargement (BPE) with lower urinary tract symptom (LUTS), age > 45 years, prostate size > 30 mL, IPSS > 7, PVR volume ≤ 1500 mL, and PSA < 4 ng/mL. Patients with PSA ≥ 4 ng/mL were included only after digital rectal examination, true-cut biopsy, and long-time follow-up visit of stabilized PSA in order to make sure without the presence of PCa. Exclusion criteria were history of urinary tract infection (UTI), previous lower tract surgery or procedures, and neurogenic bladder dysfunction. Inclusion criteria for controls included age 40–79 years, clear consciousness, ability to provide a blood sample, and willingness to complete a medical examination. Detailed information of controls was previously reported in Ma et al. [15].

 Our study population included 426 BPH patients and 1,008 healthy men. BPH cases were treated with a combined therapy of 4 mg *α*-adrenergic blockers (doxazosin) and 5 mg of 5*α*-reductase inhibitors (finasteride) once daily for at least nine months. Patients were classified into “aggressive BPH” group if they suffered from a significant increase in the IPSS score, continuous decrease in maximum urinary flow rate or BPH related complications (acute urinary retention, bladder stone or recurrent hematuria, etc.), or underwent an operation after the combined medication therapy. In contrast, patients with stable disease and without indications to receive invasive treatments were assigned to the nonaggressive group. Thus, 184 aggressive and 242 nonaggressive BPH cases were defined. 

### 2.2. SNP Selection

 A recent study reported that rs2252004 was associated with PCa risk at a genome-wide significant level in a Japanese population. This finding was confirmed in a Chinese population and evaluated in the current study. 

### 2.3. Genotyping

The SNP was genotyped for all study subjects using the MassARRAY iPLEX system (Sequenom, Inc., San Diego, CA, USA) at Fudan University in Shanghai, China. Two duplicates and two water samples were included in each 96-well plate as PCR-negative controls. All assays were performed in a blinded fashion. The overall genotyping rate was 98.5%. The average concordance rate between samples was 100% among the duplicated quality control samples.

### 2.4. Statistical Analysis

 Genotype distributions of the SNP were tested by Hardy-Weinberg equilibrium (HWE). Logistic regression was used to evaluate the association between the SNP and prognosis of BPH (BPH versus controls) and aggressiveness (aggressive versus nonaggressive) with the adjustment of age. The SNP for BPH risk was also estimated using a logistic regression model, assuming an additive mode of inheritance and with the adjustment of age. Clinical variables such as IPSS, TPV, total prostate-free antigen (tPSA), and free prostate-free antigen (fPSA) were not normally distributed and were transformed using log transformations. The changes (after minus before treatment) of IPSS, TPV, tPSA, and fPSA were normally distributed and were not transformed. Association between the SNP and these quantitative clinicopathological traits was analyzed by linear models, adjusting for age. All the above statistical analyses were conducted using PLINK software [16]. Results are expressed as odds ratio (OR) and 95% confidence intervals (CI). Minor Allele Frequency is abbreviated to MAF. *P* values were two tailed. An alpha of 0.05 was used to claim statistical significance. 

## 3. Results

The detailed clinical characteristics of all subjects were described in detail in our previous study [9, 10]. Briefly, age distribution was significantly different between cases and controls (*P* < 0.05). Therefore, all subsequent statistical analyses were age adjusted. No significant differences in clinical characteristics were found between the aggressive and nonaggressive groups (Table 1).

Genotype frequency distributions of rs2252004 were in Hardy-Weinberg Equilibrium (HWE) in both cases and controls (All *P* > 0.05, data not shown). As shown in Table 2, rs2252004 was significantly associated with aggressive BPH (*P* = 0.03). Patients who carry the risk allele “A” of rs2252004 had a 1.42 increased risk for aggressive BPH, compared with patients carrying a “C” allele (OR = 1.42, 95% CI: 1.04–1.96). Although the frequency of allele “A” was higher in BPH cases (MAF = 0.31) than in controls (MAF = 0.28), the difference in the allele frequency between BPH cases and controls did not reach statistical significance (*P* = 0.23) (Table 2).

We did not observe a statistically significant association between genotypes of rs2252004 and basic clinical characteristics, including IPSS, TPV, tPSA, and fPSA (Table 3, all *P* > 0.05). 

Furthermore, we evaluated whether rs2252004 was associated with the changes in clinical characteristics after treatment (Table 4). Results indicated that rs2252004 was significantly associated with the changes of IPSS and TPV (*P* = 0.045 and 0.025, resp.). Men who carried the “A/A” genotype (homozygous minor allele) of rs2252004 had an average increase of 1.38 in IPSS, while men carrying “A/C” or “C/C” genotypes had decreased IPSS score of 0.14 and 0.2, respectively (Beta = 1.61, *P* = 0.045). Similarly, men who carried the “A/A” genotype (homozygous minor allele) of rs2252004 had an average increase of 4.62 mL in TPV, while men carrying “AC” or “CC” genotypes had decreased TPV of 0.3 mL and 0.84 mL (Beta = 5.35, *P* = 0.025). This suggested that IPSS and TPV increased after the treatment of men carrying homozygotes for the “A” allele of rs2252004. Significant associations were not observed for rs2252004 with the change of tPSA and fPSA (*P* > 0.05, Table 4).

## 4. Discussion

BPH is a prevalent disease, especially in old men. This chronic disease has important care implications and financial risks to the health care system. Current management options for BPH include medications and prostate surgery. Taking into account adverse effects of invasive surgery and good therapeutic effect of medical management, more clinical pharmacists and patients tend to select drug therapy as a first treatment choice. At present, alpha-adrenergic receptor blockers (*α*-blockers) and 5alpha-reductase inhibitors (5-ARIs) are used for the medical therapy of BPH. The Medical Therapy of Prostatic Symptoms (MTOPS) study reported that during 4.5 years of followup, the progression of BPH, the worsening of symptoms, and BPH-related surgeries decreased more in patients prescribed doxazosin and finasteride combination therapy compared with patients prescribed doxazosin or finasteride monotherapy [17]. A long-term study (i.e., 10 years) reported that the incidence of BPH-related surgeries was only 3.2% for patients who received combination therapy [18]. So, combination therapy has been recommended as a standard drug therapy. However, in the end, there are still a number of men who require invasive treatments; this not only leads to heavy financial burden but also delays the best operation time for patients. Therefore, personalized therapeutic strategies are needed to maximize the treatment effects of medication while minimizing side effects. The genetic locus identified on 10q26 in our study may potentially serve as a molecular marker to predict individual's response to the combined therapy of BPH.

Previous studies have shown that interindividual variation in drug response is partly determined at the genetic level [19]. SRD5A1 and SRD5A2 are targets for 5*α*-reductase inhibitors. We previously reported significant associations between drug efficacy and eleven tagging SNPs (tSNPs) of these genes in a Chinese population [20]. Particularly, rs523349 (V89L) and rs612224 in the *SRD5A2* gene were statistically significantly associated with the change of IPSS and tPSA after treatment, and rs166050 in *SRD5A1* was significantly associated with the change of TPV after combined treatment [20]. However, no association was observed between the polymorphisms in the a1A-adrenoceptor genes and the treatment effect of a1-adrenoceptor antagonists [21, 22]. Therefore, additional efforts are needed to identify genetic markers predicting drug response for BPH treatment that are beyond the target genes for 5*α*-reductase inhibitors and a1-adrenoceptor antagonists.

We recently observed that several SNPs contributing to PCa risk and identified through GWAS also play an important role in BPH risk and BPH aggressiveness. Three SNPs, rs12621278 in *ITGA6* at 2q31, rs339331 in *RFX6* at 6q22, and rs103294 in *LILRA3,* were also significantly associated with BPH risk [10, 11]. More importantly, we found that rs12621278 and rs12653946 at 5p15 were significantly associated with BPH aggressiveness [10]. Although molecular mechanisms of these SNPs are unclear, this evidence supports the common influence mechanism of BPH/PCa and provides new insight into the genetics of BPH and clues about the direction of future studies. 

The SNP rs2252004 located in 10q26 was a novel locus recently identified to be associated with PCa risk in a Japanese population and subsequently confirmed in a Chinese population [12]. Our current study further showed the association between rs2252004 and aggressiveness of BPH in elderly Chinese men. Patients with the “A/A” genotype of rs2252004 responded worse to the combined therapy, compared with men who carry “A/C” or “C/C” genotypes. Interestingly, the risk allele for PCa was the major allele of “G” in Japanese and Chinese, while the minor allele “A” was the risk allele for aggressive BPH in the Chinese population. This result may reflect heterogeneity effect of rs2252004 in PCa and BPH. 

The nearest gene located on the 10q26 region is the *BRWD2 *gene, and rs2252004 was located ~234 kb upstream of the *BRWD2* gene. BRWD2 is associated with abnormal pubertal development, such as cryptorchidism, sperm defects and infertility, hypogenitalism, and sparse pubic hair [23]. This gene encodes a member of the WD repeat protein family. Members of this family are involved in a variety of cellular processes, including cell cycle progression, signal transduction, apoptosis, and gene regulation. Although the mechanism of rs2252004 underlying the association of BPH aggressiveness remains unclear, we speculated that rs2252004 may regulate the expression of BRWD2 gene through the enhancer mechanism. 

There are two limitations that need to be noted regarding the present study. Firstly, the relatively small sample size led to low statistical power to detect SNP with modest effect size. Secondly, total PSA level of 42% BPH cases exceed the 4 ng/mL. However, all these subjects received transrectal ultrasound guided prostatic true-cut biopsy to rule out PCa and vigilant follow-up observation. Further studies are needed to confirm our results in a larger sample size and with more strict inclusion criteria.

## 5. Conclusion

Although further elucidation of the biological mechanisms that influence the efficacy of medical treatment for BPH is needed, molecular genetic approaches might provide an opportunity to identify predictive markers of clinical response. In our current study, we successfully identified significant association of rs2252004 in 10q26 and aggressiveness of BPH. This result indicates an important role of rs2252004 in prostatic disease development and future clinical application value.

## Figures and Tables

**Table 1:** The clinical characteristics of all subjects.

	Cases		Controls
	*N* = 426		*N* = 1008
	Aggressive (*N* = 184)	Nonaggressive (*N* = 242)	
Age			
Mean ± SD	73.84 ± 7.97	70.45 ± 7.44	61.24 ± 8.96
IPSS			
Mean ± SD	18 ± 6.3	14 ± 6.2	N/A
PSA			
tPSA (ng/mL)			
<4%	107 (58.2)	139 (57.4)	N/A
≥4%	77 (41.8)	103 (42.6)	N/A
fPSA (%)*			
<25%	125 (67.9)	100 (41.3)	N/A
≥25%	58 (31.5)	141 (58.3)	N/A
TPV			
Median (*Q*1, *Q*3)	10 (50, 90)	74 (62, 90)	N/A

*fPSA and IPSS information from one of the aggressive BPH cases missed.

**Table 2:** Association results for rs2252004 and BPH aggressiveness/risk.

Chr/BP^1^	Genotype^2^		MAF	OR (95%CI)^4^A/A, A/C additive model	*P* value^5^
10/122, 834, 699	A^3^/C	Aggressive versus non-aggressive	0.357	1.42 (1.04–1.96)	**0.03**
			0.283		
		BPH versus controls	0.314	1.14 (0.92–1.4)	0.23
			0.279		

^1^BP: base pair; based on NCBI Build 36.

^2^Genotypes are indicated by minor/major allele.

^3^A is risk allele.

^4^OR and *P* are calculated based on logistic regression, adjusting for age.

^5^ *P*-values are based on additive models.

**Table 3:** Associations between rs2252004 and baseline clinical data.

Traits	IPSS	TPV (mL)	tPSA (ng/mL)	fPSA (ng/mL)
Alleles^1^	A/C	A/C	A/C	A/C
*β* (SE)^2^	0.02 (0.03)	−0.01 (0.03)	0.07 (0.05)	−0.02 (0.08)
*P*-values^3^	0.44	0.75	0.22	0.81

^1^Alleles are indicated by minor/major alleles.

^2^Beta and standard error results based on log-transformed data for IPSS, tPSA, fPSA, and TPV.

^3^ *P*-values calculated using linear regression, assuming additive model, adjusting for age.

**Table 4:** Effects of rs2252004 on the change of IPSS, TPV, tPSA, and fPSA after treatment in BPH case group.

Change^1^	Mean change	β (SE)^2^	*P* (Rec)^3^
	AA/CA/CC		
IPSS	1.38/−0.14/−0.2	1.61 (0.80)	**0.045**
TPV	4.62/−0.3/−0.84	5.35 (2.38)	**0.025**
tPSA	1.18/0.52/0.56	0.64 (0.69)	0.357
fPSA	0.37/0.12/0.05	0.28 (0.29)	0.335

^1^The changes of IPSS, TPV, fPSA, and tPSA were calculated using the values after treatment minus the values at baseline.

^2^Beta and standard error results based on nontransformed data for IPSS, TPV, tPSA, and fPSA for risk allele A using recessive model (CA/CC as the reference group).

^3^The *P*-values are analyzed under the recessive model with linear regression adjusted by age.

Data of tPSA and fPSA did not show (*P* > 0.05).
